# Understanding drug-related harms as *risk-amplifying loops* among people who inject drugs in Sweden

**DOI:** 10.1186/s12954-025-01267-z

**Published:** 2025-07-04

**Authors:** Jessica Storbjörk, Eva Samuelsson, Josefin Månsson, Jukka Törrönen

**Affiliations:** 1 Department of Public Health Sciences, Stockholm University, Stockholm, S-106 91 Sweden; 2 Department of Social Work, Stockholm University, Stockholm, S-106 91 Sweden

**Keywords:** Drug-related harm, People who inject drugs, Emergent causality, Needle and syringe exchange program (NSP), Actor-network theory (ANT), Sweden

## Abstract

**Background:**

Reducing risks and harms among people who inject drugs by, for example, Needle and Syringe exchange Programs (NSP) may be impeded in practice by, for example, policy restrictions, particularly in settings like Sweden where a zero-tolerance drug strategy prevails. In practice, risks and harms are produced through an interplay of multiple mutually reinforcing factors. Moreover, risk management strategies may constitute risks per se and generate new risks, potentially intensifying overall harm. This study aimed to increase our understanding of how such risks are generated in the lives of people who inject drugs.

**Methods:**

In 2022–2023, we interviewed 32 purposively selected research participants, primarily recruited through the Stockholm NSP. Drawing on actor-network theory, we analyzed the interviews to identify factors—constituent human and non-human actors—that constitute and generate risk and harm. These dynamics were conceptualized as *risk-amplifying loops*, in which harms are contingently enacted, may multiply, and the effects of policy and practice may become unintentional and unpredictable.

**Results:**

Four risk-amplifying loops were inductively elucidated: *the Service deficit*,* Perpetrator-victim*,* Deprivation*, and *Solitude loops*. In each, two actors—the *drug* and the *person who injects drugs*—were constituted differently. Furthermore, the loops were interlinked and more fully understood in relation to one another, forming a network that reflected the broader environment of injection drug use (IDU) in Sweden. Each loop was shaped and co-constituted by the prohibitionist framing of Swedish drug policy influencing access to services, the drug market, and the position of people who use drugs.

**Conclusions:**

Understanding drug-related harms as risk-amplifying loops highlights the emergent effects of the multiple and unfolding risks in the lives of people who inject drugs. This perspective facilitates discussion of impediments to effective harm reduction practices and points to potential sites for countermeasures and policy reform.

## Background

The harms of injection drug use (IDU), both for individuals and communities, are well established. In 2022, an estimated 13.9 million people worldwide injected drugs [1]. Beyond individual-level drug-related harms—such as risks of fatal overdoses and blood-borne infections [1]—social science research has offered broader insights into the risks and harms experienced by people who use and inject drugs. For instance, building on critiques of *responsibilization* and harm reduction efforts too closely focused on individual behavior, the *risk environment framework* [2] shifts attention to the situations in which drug-related harms are produced and reduced through an interplay of multiple social and structural factors [3].

International research has shown how people who sell drugs both shape and are shaped by their risk environment [4]; how law enforcement practices influence health outcomes among people who inject drugs [5]; and how HIV risk must be understood in relation to social and structural factors [3]. Certain spaces can constitute and produce risks, or enable risk-reducing processes and practices through specific social and structural conditions [6]. For instance, opioid agonist therapy (OAT) in Denmark has been found to generate both risks and enabling resources [7]. In Sweden, studies have demonstrated that the absence of adequate harm reduction services leads individuals to develop their own strategies to manage risk [8–10], sometimes resulting in avoidable risk behaviors among people who inject drugs and contributing to a “cycle of marginalization” [8].

The research project on which this study is based—*Risks of Injection Drug Use in a Swedish Context: Prevention of Harms in Practice According to Users*,* Treatment Staff*,* and Societal Actors—*centers around the Stockholm Needle and Syringe exchange Program (NSP). Although NSPs are evidence-based interventions [11, 12] and promoted by international health organizations [e.g., 13], their actual harm reduction impact may be limited if, for example, accessibility remains low [14]. Considering NSPs as “evidence-making” rather than strictly “evidence-based” interventions [15] highlights that their real-world effects cannot be known a priori, but are continually shaped as they unfold. Interventions and outcomes are locally shaped as multiple factors come together [15, 16].

This is particularly relevant in the context of this study, as Sweden has long maintained a strict prohibitionist drug policy. The introduction of NSPs was only possible in 2006 when framed as an infection control measure rather than a drug policy reform [17, 18]. Although Sweden has, since the troubling rise in drug-related deaths (DRD) in the early 2010s [19], taken steps to expand and introduce new harm-reducing interventions [18, 20], moralizing and re-educative pretenses remain dominant and continue to permeate much of Swedish society and services [9]. However, responses to DRD included that previously near-uncontested resistance to harm reduction gave way to efforts to reverse the trend—such as loosening OAT regulations (2016), legislative reforms mandating NSPs in every region (2017), and facilitating take-home naloxone (2018) [18, 20]. Still, the prohibitionist and control-oriented stance remains deeply rooted. Sweden adopted a zero-tolerance vision in the late 1970s, and since 1988, not only drug possession but also personal use has been criminalized, heavily policed, and punished with harsh penalties [21]. Recent calls by national agencies—and even the parliament—between 2018 and 2020 to review the effects of criminalizing personal possession and use have been consistently rejected by the Government [22].

NSPs in Sweden remain so strictly regulated that some stakeholders do not consider them genuine harm reduction initiatives [23]. In addition to the 18-year age limit, patients must present identification, register with the program, regularly report their drug use and risk behaviors—which are recorded in a dedicated register (inaccessible to other providers) —and consent to hepatitis and HIV testing. Staff are required to report individuals to social services if they suspect contact with minors (e.g., children, siblings, grandchildren) or if the individual is under investigation for compulsory care. NSPs also offer a broad range of services, including referrals, vaccinations, and Hepatitis C treatment [23–25]. The Stockholm NSP also provides take-home naloxone [20].

In such a context—and based on our premise that risks, harm reduction, and their outcomes continuously unfold—drug-related harms are not caused solely by individual behaviors or isolated factors. Rather, they emerge through a network of interacting factors that co-produce harm and outcomes [15, 26, 27]. This aligns with *emergent causality* [27, 28]. As Race explains, causality unfolds through how different forces come together in specific situations, often with unforeseen results [27]. At the core is Connolly’s [28] notion of emergent causality, which challenges law-like models of cause and effect as the world is “unruly in its mode of becoming” [28]. Hence, causality is “emergent” because the character of an initial action cannot be fully known before its effects materialize. Causes and effects loop through each other, forming feedback cycles between open systems [28]. In drug policy, this has been shown by Race [27], who found that sniffer dogs used to deter drug use in Sydney nightlife pushed consumption into more dangerous virtual and private spaces [see also 29]. Seeing harm as emergent helps explain how risks can spiral in risk-amplifying loops—a concept introduced in this article—as when individual strategies to manage risks escalate harm, producing unintended consequences. Research among people who inject drugs confirms this dynamic. For instance, homelessness may lead to drug use for sleep or alertness, increasing the likelihood of binge use and syringe sharing [8, 30].

The aim of this article is to analyze these dynamics to deepen our understanding of how risks and harms are generated in the lives of people who inject drugs. We identify central *risk-amplifying loops* by delineating the human and non-human actors involved and discuss how these networks impede effective harm reduction in Sweden. In doing so, the article discloses an array of entry points for reduction of harm.

## Methods

### Setting

Stockholm is the capital of Sweden, with a regional population approaching 2.5 million. Substance use services are provided by municipal social services and the regional healthcare system. Social services provide general support and ensure that individuals receive help to exit substance use problems. Healthcare services are responsible for medical treatment, which includes NSPs (established in Stockholm in 2013). At the time of fieldwork, two permanent NSP units operated, along with one mobile unit.

### Recruitment for semi-structured interviews

Research participants were primarily recruited for audio-recorded, semi-structured interviews during visits to the Stockholm NSP between August 2022 and March 2023. Interviews lasted between 20 and 70 min (median: 48 min). Purposive sampling aimed to reflect variations in age, gender, drug use, social situation, and ethnicity, roughly corresponding to the broader NSP population in Stockholm [see 31]. Inclusion criteria were being at least 18 years old and engaging in IDU. Twenty-nine participants recruited through the NSP were complemented by three participants recruited via the web page of the Users Union and day shelters. These additional interviews helped to further explore and validate reported barriers to NSP enrolment, which were also raised by current patients. As a token of appreciation, participants received a supermarket gift card worth about € 18. Written informed consent was obtained. All participants were pseudonymized in the verbatim transcripts to protect confidentiality, and fictitious names are used. NSP staff did not have access to interview materials. Interviews were conducted in Swedish; quotations presented in the Results have been translated into English with the intention of preserving participants’ original expressions as faithfully as possible.

Most interviews were conducted in a secluded room at the NSP, while others took place during walks, in cafés, or at a day shelter—wherever the participant felt most at ease. A typical interview began with questions about the participant’s social situation, followed by their motives for and experiences with visiting the NSP. This naturally transitioned into discussions about drug use and IDU practices. The third part of the interview focused on risks and strategies for managing them. This was introduced by asking, “What does a typical day look like when you are actively injecting drugs?” Participants were then asked to contrast such a day with their own narratives of “good” and “bad” days. The interviewer probed into risks mentioned and asked participants what they considered most risky about IDU.

### Participants

The median age among the 19 men and 13 women was 45 years (range 26–64). Most participants reported using multiple substances (*n* = 7), with amphetamine being the most commonly preferred drug (*n* = 19), followed by opioids (*n* = 6)—typically heroin, buprenorphine, and methadone. Participants were categorized according to their social situation as either “marginalized” (lacking income and stable housing, *n* = 10), “welfare recipient” (receiving needs-tested social or financial support, *n* = 16), or “integrated” (having employment, receiving pension or sickness benefits, and stable housing, *n* = 6). While most identified with Sweden’s majority population, nine participants were categorized as belonging to minority groups (e.g., ethnic backgrounds in Roma communities or in South America, Africa, the Middle East, or other Nordic countries).

### Initial open coding

Six members of the project read all interviews before convening for a full-day discussion to develop a tentative coding tree grounded in the project’s research questions, theoretical framework, and empirical observations. Next, to ensure solid interpretations and intercoder reliability, JS, ES, and JM initiated joint line-by-line open coding to further refine the coding tree in a theory- and data-driven manner. Five interviews were jointly coded using the NVivo software, followed by individual coding of three to five interviews each. The team reconvened to code one more interview together before finalizing the open coding.

For this article, two broad codes were extracted, covering participants’ explicit and implicit accounts of *risks* and harms in their lives, and *strategies to manage these* (≈ 183,000 words). It was important to also include latent content, as participants might, for example, mention in passing that their partner had overdosed from adulterated drugs without explicitly naming it as a risk.

### Actor-Network theory (ANT) as analytical tool

The two codes revealed a wide range of articulated risks encompassing health, social, economic, and legal dimensions. Structural factors, such as the zero-tolerance drug policy, were noticeably entangled with individual-level risks like staphylococcal infections or damaged veins. Moreover, contextual or structural interpretations often lack direct data, as they are typically inferred from what participants share in interviews [32]. Thus, a “flatter” approach that does not impose a hierarchy between macro and micro levels was suitable for our empirical analysis.

The analysis focused on identifying *risk-amplifying loops*—understood as processes where multiple reinforcing factors come together, and where efforts to manage risks translate into or intensify new risks. Drawing on Actor-Network Theory (ANT) [16, 33–39] allowed us to map and trace how various human and non-human actors within these loops, understood as actor-networks, and as outlined in the narratives of people who inject drugs, became entangled and unfolded in practice. JS and ES applied an inductive, iterative approach similar to that described by Neale [40] to the extracts of each participant. They noted key actors and typical sequences, events, or relationships that moved the network along and amplified risks. The focus was on “what happened” in the narrative, how the loops unfolded, relational aspects, and what each actor “did” and how it made a difference in the risk production. The analysis progressed to delineating and conceptualizing these into distinct risk-amplifying loops that captured the empirical material. The last step involved outlining and illustrating interconnections between and combining the four loops into an overall actor-network of risk generation in the lives of people who use drugs in the studied prohibitionist setting.

To specify further, “actors” were understood as socio-material human or non-human components that co-constitute and exist in relation to one another in a “network” consisting of interconnected actors. Including both human and non-human actors means giving equal attention to people, things, and structures—such as *diagnoses*, *the judicial system*, *individuals*, and *socio-material resources*. Actors of interest were those that temporarily became associated with a specific loop and made a traceable difference in the risk generation of that loop. Moreover, the connections or translations changed the character and capacities of the actors. This emphasizes how actors and the network continuously shape and reshape each other as they interact. This was, in this analysis, especially clear in how the *drug* and the *individual using it* acquire different roles and capacities in each loop. For example, in the first loop (which centers on *service deficits* and the role of the negligence of care providers in risk generation), people who inject drugs are referred to or choose to manage risks on their own—becoming *self-managing individuals* who use drugs as self-care or to cope with problems that the treatment system fails to address, bringing with it amplified risks. In this network, the substance acts as a *remedy drug*. This can be contrasted with the final loop, which centers on solitude. Here, the *stigma* surrounding *drugs* turns people who inject drugs into *marked individuals*: the visible signs of substance use contribute to individuals withdrawing and keeping to themselves. In this loop, the *drug* acts as a loyal *companion*.

## Results

The four identified risk-amplifying loops are richly described below. Evident variations by socioeconomic status, gender, or age are explicated.

### Service deficit risk-amplifying loop

The Service deficit risk-amplifying loop was co-constituted of the following actors whose meaning varied depending on how they became related to other actors: inaccessible and rigid services in the form of *healthcare* (including dependence care and psychiatry), *social services*, and *the NSP; diagnoses; self-managing individual;* and *remedy drug*.

Health care became a deficiency in this loop in connection to participants’ repeated stories of how their medical and psychiatric diagnoses were inadequately addressed by care providers. In response, the self-managing individual engaged in self-care or coped with this by means of substances that acted as a remedy drug. Common risks in the interviews related to neuropsychiatric conditions—particularly attention deficit hyperactivity disorder (ADHD) mentioned by 15 participants—along with psychiatric disorders (bipolar disease, post-traumatic stress disorder (PTSD), depression, anxiety, sleep deprivation), and somatic issues like chronic pain). These diagnoses translated into specific and troubling kinds of actors in this loop, as many participants had not received formal diagnoses due to long waiting lists or unmanageable abstinence requirements during assessments, as in Thomas’s case:

I want [AHDH medication], because it works. But I’ll never get it, since I’m abusing amphetamine […]. I want it now, not after being sober for three months, because that doesn’t work. Not if you don’t have a permanent place to live. It’s impossible […] A fucking Catch-22. (Thomas, marginalized, 40–49 years)

Others were discharged after missing appointments—often due to their disability or unstable life situation. Those who had received diagnoses often avoided seeking medical care, as the demands placed on them were considered too burdensome. The imminent risk of suddenly being “cut off” from prescribed medication was considered too great:

I could get [ADHD medication] on prescription, but I can’t handle the fuss. I could be using legally […] the control. Like “Well if you don’t behave, we’ll remove it” and just cut me off […] As a user, I have to dosage my own drug because I feel… I know what I need. (Linda, integrated, 40–49 years)

Similarly, Annika (a 40–49-year-old welfare recipient) had her “medication halved” despite adhering to the doctor’s prescription, due to distrust from healthcare. This led her to “cease contact with the doctor and start using [amphetamines] illegally instead.” In this loop, participants were translated into self-managing individuals who injected drugs to manage health issues not adequately addressed by inaccessible yet paternalistic services. This is clearly illustrated by Jorge, who described how he took matters into his own hands and, as a form of self-care, used amphetamine to manage daily functioning in the absence of prescribed stimulants for his ADHD.

When my day starts it’s like throwing a plate to the ground. Like shrapnel everywhere. To get the pieces back together, to make the plate whole again, I need amphetamine. Without it, I can’t start my day—I can’t think, I can’t plan, I can’t get things done […] It’s so fucking sad I can’t get medication. (Jorge, marginalized, 40–49 years)

When Jorge anticipated that he would not receive adequate and individualized help from healthcare services, he was steered away from legal stimulants for ADHD toward an illegal approach. Rather than becoming a patient, Jorge’s self-care constituted him as a self-managing individual, using drugs as a remedy. Hence, this loop pushed him toward the illegal drug market, thereby amplifying his exposure to risk.

In a similar vein, when healthcare failed to address somatic pain, participants could manage this by turning to opioids or cannabis. Matti (a 50–64-year-old welfare recipient) used pot to counter the constant pain caused by police violence. Per turned to heroin after experiencing inadequate care related to opioid over-prescription and premature discharge (which was raised as a risk also by other participants, e.g., Samuel, 40–49 years, welfare recipient).

My addiction comes from not getting help from somatic care when I had cramps and pain […] I got Oxycontin for some years […] Based on findings from the US […] they wanted to lower my dose by half […] I admitted myself to dependence care and told them I wanted to quit […] It was like hell. The pain came back, and the abstinence and all […] The hospital just said “Well done” for quitting and shut the door […] My [partner] and mother helped me get back on Oxycontin from the online black market […] Then I calculated that heroin would be much cheaper. (Per, marginalized, 50–64 years)

With extensive histories of medical, psychiatric, and substance-related issues, participants had broad experience with the healthcare system. However, the overwhelmingly negative previous encounters often led to avoidance and self-management. For some, this meant rejecting OAT, which was perceived as overly controlling and stripping individuals of their agency—like in Rodrigo’s case (40–49 years, welfare recipient). For others, like Elin, it meant withdrawing entirely from seeking medical care due to the imminent threat of coercive care:

With the threats of coercive care, I avoided all kinds of healthcare […] I could not go to primary care or the hospital […] I had a lot of staphylococcus and stuff, and I noticed it wasn’t such a good idea to go to the emergency room or the hospital because it was like “We’ve received this fax from the social services [handling compulsory care]”. So, I just, “goodbye” […] It didn’t matter that I had crutches and people just “you are going to lose your leg”. (Elin, marginalized, 26–39 years)

Amphetamines, cannabis, and benzodiazepines were also used as remedies to cope with a lack of financial resources, homelessness, and isolation—issues typically addressed by social services. In this loop, social services were constituted as a rigid bureaucracy, which again, transformed the potential client into a self-managing individual, relying on drugs to cope. Participants described constant struggles to access the help they were entitled to. Julia (welfare recipient, 26–39 years) explained: “You have to prove so damn much, you don’t get any […] It’s just those statistical forms and meetings. So much fuss, you must fight for everything.” Financial aid and housing assistance were often conditioned by abstinence or treatment, conditions many—including Julia—found unmanageable, hence pushing participants further into the margins and greater risks.

In contrast, the NSP was portrayed as a humane place with kind staff. Many turned to the NSP for issues way beyond its intended mission [see 41]. Still, the self-managing individual remained relevant in connection to the NSP, as the strict regulations prevented some from attending. Barriers such as long distances and limited opening hours especially affected socially integrated and employed participants. They feared being seen at the NSP, its vicinity, or nearby OAT services. Hans, for example, explained he had too much to lose if recognized there.

There’s too much stigma around this, I would rather die than tell anyone […] If I went to [NSP], I’d be visible. Someone could see me […] I can’t be seen there! […] It’s that simple. So, I buy my own equipment [online]. They’re clean, work, and the small cost is worth it. But it would be nice to have a care contact, someone to share everything with, but you can’t […] Then all my prescribed medications would disappear […] Just like that. (Hans, integrated, 50–64 years)

Beyond such stigma, the geographic location and NSP’s setup—which involved forced interaction with other people who inject drugs—also deterred participants. Moreover, many feared the risk of arrest, as police often drove by, discouraging them from visiting the NSP.

Another group avoiding the NSP were those with custody or visitation rights to children, as enrollment risked losing such contact if reported to social services. This risk was especially pronounced for women. For example, Johanna, a 40–49-year-old marginalized woman, only registered at the NSP “the day I completely gave up custody of my children.”

In conclusion, participants managed the risks by avoiding becoming associated with the strictly regulated and potentially stigmatizing NSP and its visitors. Instead, they faced amplified risks—ordering equipment online (a criminal offense), sharing or reusing needles, sharpening needles themselves, or relying on others to fetch supplies. Hans, who had never visited NSP, even preferred the risk of dying over seeking care:

I’ll probably make a mistake [injecting] at some stage. Something goes wrong. I’d probably have to visit healthcare […]. No, I won’t. I know that. I’d rather die at home. (Hans, integrated, 50–64 years)

While the remedy drug in this loop helped participants maintain their integrity and cope with difficulties, such as suicidal thoughts, the effects of this loop obviously entailed new risks, including infections and becoming involved in criminalized, marginalized lifestyles.

### Perpetrator–victim risk-amplifying loop

In the second risk-amplifying loop, the substance acted as an *illicit drug*, co-constituted by the *judicial system* (police, courts, prison) and its extension in *criminal records and debts*, *private* *security officers*, the *drug market*, and the alternations of a *perpetrator-victim individual*. The meaning of these actors changed depending on their connections within this loop.

Since selling, buying, possessing, and using drugs are criminalized, in this actor-network the person who injects drugs constituted a perpetrator by the judicial system. In embodying this role and dealing with accompanying risks such as getting arrested, participants often found themselves in even riskier situations and translated into victims, for example, if peers did not call for an ambulance in overdose situations out of fear of arrests since the police often accompanied ambulance staff. In this loop, the illicit drug and the perpetrator-victim individual were shaped by their relationship to the judicial system and the co-constituted drug market.

One way this loop could unfold was when private security officers—and sometimes police—confiscated injection equipment and drugs, amplifying the risk of sharing or reusing needles and syringes.

Several times the police have taken… I understand that they take the drugs, but not the clean tools—unused packages. And I think, ”Seriously? Come on, you know I won’t stop just because you take this away.” […] It shocked me that the police would do that. (Elin, marginalized, 26–39 years)

In our sample, there was a shared understanding that the police—though sometimes friendly—constantly chased people who use drugs, aiming to make drug use undesirable. As Johan (welfare recipient, 26–39 years) put it, “they hunt us down with a blow torch” and “want to disturb as much as possible, but the only thing they do is to destroy people’s lives more.” The frequent police presence near NSP and OAT services, including body searches and confiscations, was described as a means to prevent people who inject drugs from attending and pushing them to share injection equipment.

Participants noted that successful police raids created shortages in the drug market. To avoid withdrawal, they were forced to take or mix the only substances available—despite drugs risk being adulterated with fentanyl—leading to further harm or even death. Drug shortages also drove some to try alternative substances, which carried new risks. For example, during a heroin shortage, Per turned to amphetamines and became attached to that drug.

When corona came […] there was no heroin for me that day, and I was with two friends who were using amphetamine […] They said “Take this. You are sick”. I said, “But I’ll feel even worse”. […] But then I was like “why did nobody tell me this?” It was wonderful. I fell in love instantly. (Per, welfare recipient, 50–64 years)

Although many participants had stable suppliers, the drug market in this loop was marked by the immanent risks of adulterated substances and violence. Both selling and buying drugs required resources like trust—and, ultimately, violence. Age and gender shaped these risks: older men, for instance, might lose their previous “violence capital” and had to compensate with weapons or rely on physically stronger companions. Involvement in drug selling also carried the risk of overuse due to easier drug access.

Participants described the effects of this loop as a “sticky web” that obstructed recovery and blocked access to work and housing. Even those on legal opioids through OAT testified to continued harassment by private security officers, such as being thrown off the metro. Debts from unpaid drug-related fines, handled by the Enforcement Authority, and criminal records made it difficult to secure housing or employment. Progress was also obstructed when the judicial system denied alternatives to prison, such as electronic monitoring, which made Jorge (marginalized, 40–49 years) miss a welcomed job opportunity.

In these processes, the involvement with the illicit drug positioned the individual as a perpetrator, and in trying to manage the consequences, the person often became captured as a victim of the resulting circumstances.

### Deprivation risk-amplifying loop

Several actors came together in the struggle to manage daily life and basic needs amid scarce resources. In the Deprivation loop, the *individual in need* was co-constituted through *limited socio-material resources*, *technology*, the *absence of or forbidden spaces*, an *erratic drug*, the “*user collective”*, and *violence*.

This loop diminished capacities for well-being and was especially apparent among participants who lacked basic resources such as income, food, shelter, and a safe space. These conditions were partly shaped by the difficulty of accessing support from social services, leaving marginalized individuals in need to fend for themselves within risky informal economies and social settings.

The lack of housing and support systems forced participants into a constant chase for a place to sleep, shower, store belongings, access food, and charge phones—illustrating how the individual in need was constituted by the absence of safe spaces and socio-material resources. Both the deprivation itself and the daily chase were associated with extensive risks. Technology further amplified difficulties: without a phone, participants became more exposed, lost access to help, connection, and the ability to buy drugs—turning the substance into an unpredictable source of relief with no guarantees of comfort: an unpredictable, erratic drug. Not having a digital ID (linked to phones) excluded participants from most contacts with authorities. Even with money in the bank, access could fail due to technology (no digital ID) or lack of permanent address, which prevented, for example Jan, to receive the pin code for his debit card: “It’s really frustrating when you have money in your account but you can’t do anything. I can’t even buy food because I don’t have the code.” (Jan, welfare recipient, 50–64 years).

Squatting in abandoned buildings exposed participants to mold, fire hazards, and harassment from private security officers in such forbidden spaces. Lacking basic amenities like a refrigerator, participants often had to steal food daily, increasing the risk of getting caught. To avoid the gaze of the public, some chose to sleep rough—risking hypothermia—or sought refuge in other forbidden and unsafe spaces like stairwells or public toilets, sometimes sharing these with a violent partner.

Even when granted supported housing by social services, participants often avoided these inhospitable places. Some preferred sleeping in attics over staying in places described as “more or less brothels, or crack houses” (Elin, marginalized, 26–39 years).

I used to stay in a shelter, but had to share room with bad people who didn’t respect others’ need to sleep. I stayed awake until they left the room. I had to, but couldn’t function during the day. (Nikos, marginalized, 26–39 years)

The staff could also be hostile and stigmatizing, “The staff is treating me really badly. They harass me”, said Anita (welfare recipient, 50–64 years). Participants also feared being caught using drugs in such housing settings. Conversely, for those trying to quit, low-threshold accommodation was a risky option, as abstaining was difficult around others actively using drugs.

When forced to share space with others in similar situations, friction often arose within the “user collective”, where companions could shift from friends to foes. Participants described repeated experiences of being robbed of phones, injection equipment, drugs, and other belongings. The interviews also recounted everyday violence, rape, and jealousy in these oft-involuntary social settings. Regardless of trust or affinity, participants frequently depended on others for drug transactions or crimes to obtain necessities or substances. Some used drugs to cope with or dare to commit these crimes—illustrating how risks unfolded in this loop. Managing this life was often seen as harder for women: “The girls here are tough. You have to be hard, and tough, and yell, and fight, if you’re going to make it in this situation” (Karin, welfare recipient, 50–64 years).

Being exiled to public spaces amplified risks, particularly when moving through the city, often via public transport. Without money for tickets, participants faced fines or being thrown out of this forbidden space. Lacking safe places to store drugs, they risked being caught for possession if noticed by the police. Even private security posed a risk:

The guards […] they smash people all the time. […] You get beat up. Once I bought a phone. […] Three days later the guards came and started talking. […] It ended up with them taking me to some room beating me up. […] One of them put the phone on the floor and just smashed it. […] Those guys are lethal. (Samuel, welfare recipient, 40–49 years)

Violence was a constant actor in participants’ stories—fear of being killed, stabbed, or needing to arm oneself, which in turn risked long prison sentences, as in Lars’ case:

I was stabbed twice in the back, up by the neck, and one of my lungs was punctured. […] It takes more than that to kill me […] I’d call some of my old good friends—bigger and much more mobile than me—to come and sort things out. For a while, I had a shotgun. […] But I’m not looking to do life in prison for some fucking idiot who can’t stay calm. But if it came to that, I wouldn’t hesitate for a second. My life is worth more than theirs. Simple as that. (Lars, welfare recipient, 50–64 years)

In this loop, individuals in need, deprived of basic necessities, often relied on substances in everyday life: to sleep in the cold, stay awake while roaming without shelter, or find the courage and energy to commit crimes, etc. (e.g., Johanna and Annika).

Try staying [sober] when you don’t know where you’ll wake up in the morning, and nothing’s prepared [drugs]. It’s nearly impossible […] Sometimes there’s nowhere to sleep […] I have to stay awake. Well, amphetamine works pretty well then. […] It numbs your feelings too. (Emma, marginalized, 26–39 years)

Yet, as explained, although substances could offer comfort, the volatile life and uncertain drug supply translated substance into an erratic drug in this loop.

### Solitude risk-amplifying loop

Although the life described above could seem unbearable, some participants chose it to avoid complete loneliness.

That’s what’s difficult when you quit [drugs…]. You get lonely. […] What’s the point of quitting then? […] Now when I’m active I don’t have to be lonely all the time because I’m doing drugs. I go out and meet people. (Samuel, welfare recipient, 40–49 years)

In this final loop, life in *solitude* was the only conceivable way to manage the risks of IDU. The meaning of *solitude*,* a marked individual*, the *companion drug*, the *stigma* attached to drugs, and the “user collective” was shaped in relation to participants’ level of social integration or marginalization.

To avoid risks like becoming associated with deprivation, the “user collective”, or the police, some more marginalized individuals marked by IDU kept to themselves and connected with solitude. This created a loop where loneliness brought severe risks, including depression, suicidal thoughts, and a higher chance of fatal overdose from injecting alone. Viktor, who tried to avoid the “user collective” to steer clear of crime, illustrates this:

I never interact with [other NSP visitors], I use alone, so I’m completely alone, which has its ups and downs. It’s sort of good because I’m not drawn into more criminality, but negative when it comes to the risk of overdose. (Viktor, integrated, 26–39 years)

Solitude thus manifested in contradictory ways. Those trying to break away from harmful company in the “user collective” sometimes “slip because they cannot handle the loneliness” (Marko, welfare recipient, 26–39 years). To avoid loneliness and its risks, some formed destructive partnerships that made staying off drugs harder.

I’m planning to leave him and go to [another town] because I really don’t want to keep living like this. All I want is to smoke cannabis. […] Even though I care about him, I can’t [be with him], it’s too destructive, too much fighting. It’s harder for me to stay off [drugs] when he’s using. (Karin, female welfare recipient, 50–64 years)

This loop took a different form among more socially integrated participants. Because of the severe stigma around IDU, these individuals—who were marked by IDU but not yet part of the “user collective”—lived in fear of being identified as people who inject drugs. To avoid stigma, shame, registration as drug users, and contact with other people who inject drugs, they chose not to enroll in the NSP (e.g., Marko, Maria). Instead, they isolated themselves to keep their drug use secret (e.g., Hans). They could usually afford to buy injection equipment online, which, however, involved criminal risks.

Those connected to the labor market faced added challenges: they feared colleagues discovering their use and struggled with daily OAT appointments conflicting with work, increasing risks of loneliness, relapse, or heavier use (e.g., Viktor).

I’m on sick-leave for another month, because I switched to methadone from buprenorphine, which didn’t suit me […] The thing is, my boss doesn’t know about my drug use. I have to go every day to get my methadone during their opening hours. It’s hard trying to find something to say to [boss], where I’m going every day, to get my medicine. But if I behave at the start [of OAT], I’ll be allowed to take home doses. Of course they want me to keep my job. They want to support me if I can show them I’ll behave. (Sara, integrated, 26–39 years)

In this loop, the drug was experienced as a friend—a stable companion in solitude. Participants generally did not express a desire to give up this companion. Those with stable housing described detailed, even sensual, injection rituals, where risk management was more feasible thanks to better access to resources.

However, the “sticky web” of stigma persisted. Participants explained that the visible marks of injecting—scars, bumps, damaged veins—made it impossible to be honest with bosses or social contacts over time. These signs continued to affect their social lives, even when they were drug-free or trying to get their lives back on track.

### Interconnecting the risk-amplifying loops

**Fig. 1:**
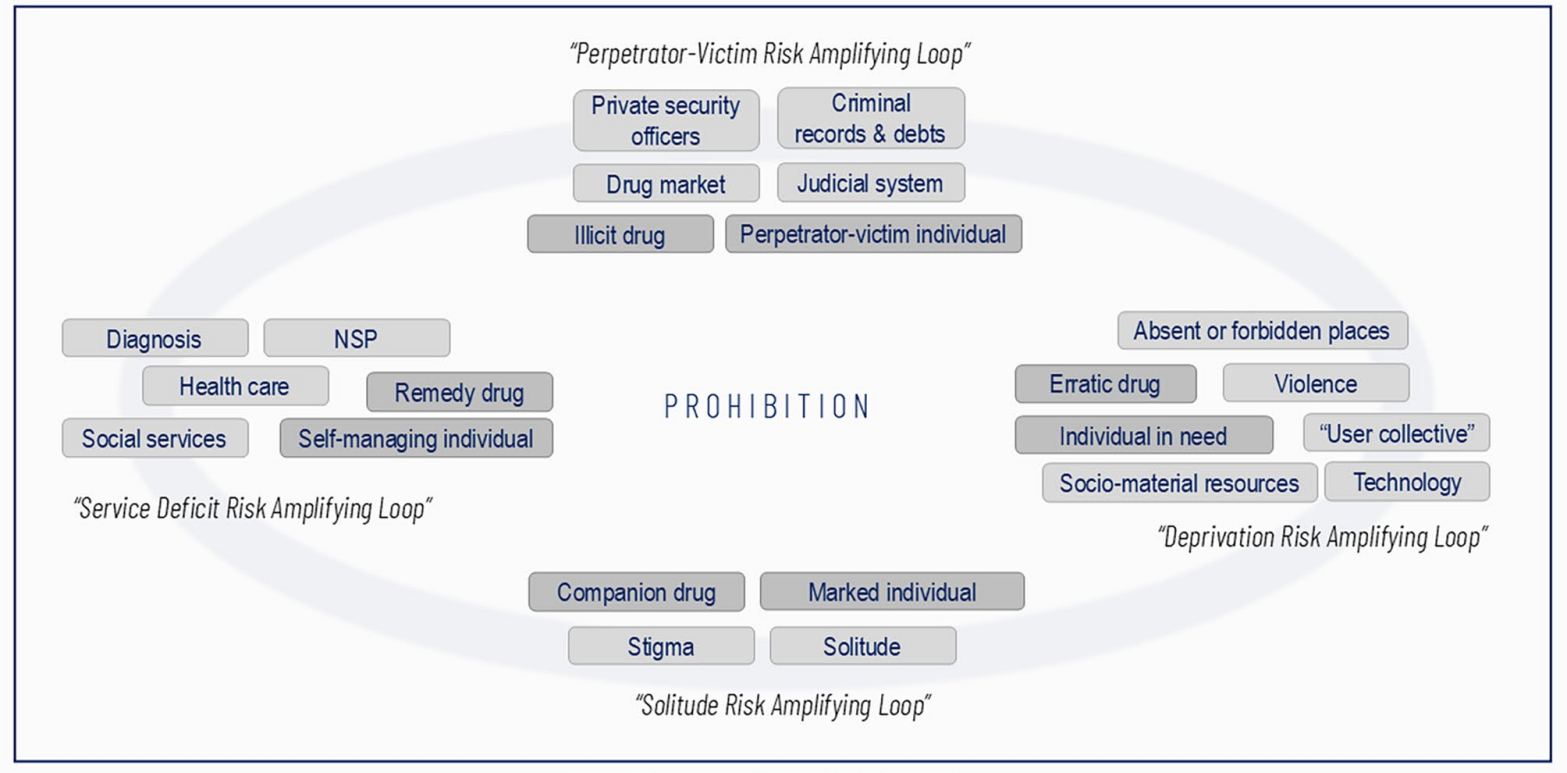
The interconnection of risk-amplifying loops in the lives of people who inject drugs

The four risk-amplifying loops were connected, forming a broader actor-network that caused harm among people who inject drugs. Although clearly delineated loops, in which actors such as the *individual* and *drugs* acted in certain ways, several links between loops were noticeable, as evident above. Considering each loop as relevant to other loops and linking certain actors across loops added to our understanding of emergent drug-related harms (Fig. 1) [27, 28].

The concrete characteristics of the actors in the networks were shaped by each risk-amplifying loop and the relationships by which they were translated within the loop. The figure illustrates how some actors can gain different capacities as they become part of and interact within a larger actor-network [36, 38]. Most notably, the individual and the drug were characterized differently by their in-loop translations. The multiple individuals (as self-managing, perpetrator-victim, in need, and marked) enabled varied relationships with the multiple forms of drugs (remedy, illicit, erratic, and companion). Emergent and amplified risks arose within and from these links.

For example, we noted how social services in the Service deficit loop tied into the lack of socio-material resources in the Deprivation loop. Similarly, deficits of the NSP (also the Service deficit loop) connected to the “user collective” in the Deprivation loop, which in turn tied into stigma and solitude. The spaces, violence, and “user collective” were further constituted by the drug market and the Perpetrator-victim loop.

All four loops were formed in close connection with the zero-tolerance drug prohibition in Sweden. The Perpetrator-victim loop was rooted in the criminalization of selling, buying, possession, and personal use. This influenced the Deprivation and Solitude loops, by transforming relationships among people who inject drugs and their interactions with society—e.g., stigma, and conditioning of services. Moreover, strict regulation of NSP was the only viable option to combine harm reduction with prohibition policy [17].

## Discussion

This article has explored how risks in the lives of people who inject drugs may spiral and intensify when the strategies intended to manage risk are themselves inherently risky. Similar dynamics have been previously observed in attempts to balance competing risks. For instance, avoiding arrest may increase the danger of not being found during an overdose if the person hides in a secluded alley [42, 43]. Likewise, navigating medical, administrative, and dosage restrictions in OAT may drive people toward the more hazardous illegal drug market [7]. However, our conceptualization of these processes as *risk-amplifying loops* emphasizes the compounded nature of risk for people who inject drugs—how risk management strategies can generate further risk through *emergent causality* [27, 28].

The identified *Service Deficit*, *Perpetrator-Victim*, *Deprivation*, and *Solitude* risk-amplifying loops uncovered demonstrate how people who inject drugs in Sweden frequently encounter systemic “dead ends”. In these loops, key institutional and social components are configured in ways that facilitate certain behaviors—often risky or harmful—while obstructing access to healthier alternatives [26]. Through these processes, people who inject drugs become increasingly detached from social networks that support stable housing, employment, and family life. As Latour [16] observes, emancipation is not about being “freed from bonds”, but about being “well-attached” [see also 39]. In other words, individuals require robust networks of attachments to act effectively, solve problems, and navigate everyday challenges. These four loops illustrate how participants progressively lose the social and material ties essential to living a “normal life”, thereby diminishing their life-sustaining attachments.

These systemic dead ends manifest, for example, when people who inject drugs in a service-deficient system are unable to obtain diagnoses or adequate treatment. Left to manage their issues independently, many are funneled into the illegal drug market—reframed as perpetrators or victims, rather than patients deserving of care. Similarly, buprenorphine and methadone are commonly regarded as substitution “medicines” that enable people to avoid illicit drug use and lead more stable lives. However, ambivalence toward these treatments may reflect the trade-offs imposed by institutional constraints, including clinic locations and restrictive operating hours [7], which among participants imposed clear barriers to access services irrespective of resources [see 41].

Rhodes [2] has argued that environmental conditions not only influence the adoption of harm reduction policies but also shape their implementation and impact. In Sweden, emergent effects [27, 28] of the broader risk environment [2, 3] are evident in the quite recent introduction of harm reduction services such as NSP. While these programs are evidence-based and internationally recognized for reducing risky behaviors [11–13], Sweden’s highly regulated implementation of NSPs has paradoxically introduced new risks. For example, the requirement for personal identification increased vulnerability for women with children and for people maintaining partial ties to conventional society—those not yet publicly stigmatized for IDU. These contradictions are not unique to Sweden. In Denmark, for instance, OAT may offer both material support and a sense of agency while simultaneously undermining autonomy through pervasive surveillance [7].

However, as noted, all risk-amplifying loops were shaped by—and are best understood in relation to—Sweden’s zero-tolerance drug policy. The constraints highlighted in the *Deprivation* loop are closely linked to the *Service Deficit* and *Perpetrator-Victim* loops, as well as to the broader policy context. However, the drug policy impact is difficult to compare across contexts, and similar findings have been reported in other parts of the world. Research in Canada shows how people who use drugs subjected to residential eviction experience “cycles of instability”. In response to institutional demands and structural vulnerabilities, some choose to sleep rough rather than endure the chaos and surveillance of available shelters [43]. Similarly, studies from Australia underscore the co-constitution of drugged bodies and urban spaces, particularly among women [44, 45]. In these contexts, women who use drugs may face a stark binary: either becoming a “junkie” of open space or escaping them in secluded spaces with highly risky implications [44].

Beyond spatial dynamics, public scrutiny and police presence are also central to these environments [44, 45]. Our findings further suggest that an essential component of such spaces is exposure to, or association with, other people who use drugs. While some interviewees recounted supportive relationships within the “user collective”, many also described significant risks, leading some to social isolation. The Danish study of OAT clients echoed this pattern: many people avoided others due to distrust, fear of violence, or theft [7]. As Jakobsen et al. [7] noted, isolation as a coping mechanism creates new dilemmas by cutting off access to the material, emotional, and social resources that a broader network can provide.

Hence, the findings must be contextualized within the overarching stigma and socio-material deprivation that characterize life for many people who use drugs in Sweden. Prohibitionist policies and law enforcement practices push people to the margins, weakening internal solidarity among the “user collective” and impeding joint efforts to improve conditions. User unions have become less influential, and research indicates that people who use drugs in Sweden often feel more isolated than their counterparts in Danish “hot spots”, where low-threshold services are more available and community and friendliness are more pronounced [9]. In the Swedish context, with its historical roots of a paternalistic treatment system and prohibitionist drug policy [46, 47], social exclusion becomes more entrenched, and pathways out of drug use more elusive—mirroring the “sticky webs” referenced earlier.

Emergent causality [27, 28] is perhaps most visible in the effects of law enforcement. Police practices are well-documented barriers to calling emergency services during overdoses and can undermine harm reduction initiatives when officers patrol these sites, thereby increasing the risks for those who access them [48]. As Collins et al. [48] and Race [27] suggest, targeted policing of “hot spots” not only displaces drug-related activity but also intensifies precarity. Individuals in these areas face chronic uncertainty about when “street sweeps” will occur or whether their belongings will be confiscated [43]. While the efficacy of such policing has long been debated, there is growing consensus that it exacerbates health and social harms [49]. The *Perpetrator-Victim* loop in our study powerfully illustrates this: not only are people who inject drugs vulnerable to criminalization and incarceration, but this loop—emblematic of prohibitionist policy—exerts long-term influence. Moreover, it interconnects with and reinforces the other risk-amplifying loops, perpetuating the broader cycle of harm.

### Limitations

The study centered around the NSP, which means that experiences of people who inject drugs choosing not to enroll may be less reflected. These are, in part, covered by participants’ accounts of their decision to enroll and by those recruited via other sources. We were unsuccessful in recruiting participants under 25 years of age, and the sample would be strengthened by a better representation of minority groups.

While ANT has been applied to focus groups [33] and autobiographical data [37], it may be less suitable for interviews [38]. Interviews tend to prioritize human actors, filtering data through the researcher and interviewee, potentially overlooking detailed interactions with non-human actors in the network. However, our strategy of eliciting elaborations from participants about typical, good, and bad days when using drugs proved successful. Interviews yielded rich descriptions, illustrating how a “good day” involved non-human elements such as quiet morning walks, secluded paths, sitting on cardboard, and experiencing the drug in sunlight. The interviews also highlighted narratives of materialized pleasure [e.g., 50], matters of care (analysis under review) and positive encounters with the NSP [41], though this article discusses risks with minimal reference to such positive aspects. In conclusion, the interviews yielded tangible actors and translations as interviewees connected themselves to various events, individuals, and organizations [16, 33]. We judged the material to provide a robust basis for empirical analysis, mapping acting actors in the data [see 38].

## Conclusions and implications

The study contributes to a deeper understanding of how various factors—or actors—together multiply and reinforce harms in the lives of people who use drugs. By conceptualizing these processes as risk-amplifying loops, the study highlights how risks emerge and escalate as strategies for managing risks paradoxically amplify or introduce new forms of risks.

The study identified the *Service deficit*, *Perpetrator-victim*, *Deprivation*, and *Solitude* risk-amplifying loops, which were all formed in close connection with Sweden’s strict zero-tolerance drug policy.

In considering implications for harm reduction, from this ANT-perspective, prevention efforts should target the entire actor-network. Rather than isolating certain aspects—like risk behaviors of certain individuals—the focus should be on the harmful associations between actors—such as how service systems shape capacities of people who use drugs. The aim is to change how these actors influence one another when they come together [26].

Given this, many opportunities and entry points for reducing risks can be identified. Lowering service thresholds, increasing flexibility, and removing abstinence requirements could help people who inject drugs access support without being pushed into potentially harmful roles (e.g., self-management, perpetrator-victim). Creating safer spaces—both socio-materially and in relation to security and policing—could reduce solitude, and support well-being and a sense of community. These efforts, however, are often undermined by the criminalization of drug use. Still, as actor-networks are dynamic and shaped by their components, change is possible. Risk environments can become enabling ones [6, 35].

## Data Availability

The interview datasets generated for the research project are not publicly available due to the qualitative nature of the data, the potential for individual privacy to be compromised, and the procedures described to participants in the informed consent form, approved by the Swedish Ethical Review Authority.

## Abbreviations

ADHD: Attention deficit hyperactivity disorder
ANT: Actor-network theory
DRD: Drug-related deaths
IDU: Injection drug use
NSP: Needle and syringe programs
OAT: Opioid agonist therapy
